# Managing depression in people with multimorbidity: a qualitative evaluation of an integrated collaborative care model

**DOI:** 10.1186/s12875-015-0246-5

**Published:** 2015-03-05

**Authors:** Sarah E Knowles, Carolyn Chew-Graham, Isabel Adeyemi, Nia Coupe, Peter A Coventry

**Affiliations:** NIHR School for Primary Care Research and Manchester Academic Health Science Centre, University of Manchester, Manchester, M13 9PL UK; Primary Care and Health Sciences, University of Keele, and NIHR Collaboration for Leadership in Applied Health Research and Care West Midlands, Keele, ST5 5BG UK; NIHR Collaboration for Leadership in Applied Health Research and Care Greater Manchester and Manchester Academic Health Science Centre, University of Manchester, Manchester, M13 9PL UK

**Keywords:** Depression, Collaborative care, Psychological therapy, Integrated care

## Abstract

**Background:**

Patients with comorbid depression and physical health problems have poorer outcomes compared with those with single long term conditions (LTCs), or multiple LTCs without depression. Primary care has traditionally struggled to provide integrated care for this group. Collaborative care can reduce depression in people with LTCs but evidence is largely based on trials conducted in the United States that adopted separate treat to target protocols for physical and mental health. Little is known about whether collaborative care that integrates depression care within the management of LTCs is implementable in UK primary care, and acceptable to patients and health care professionals.

**Methods:**

Nested interview study within the COINCIDE trial of collaborative care for patients with depression and diabetes/CHD (ISRCTN80309252). The study was conducted in primary care practices in North West England. Professionals delivering the interventions (nurses, GPs and psychological well-being practitioners) and patients in the intervention arm were invited to participate in semi-structured qualitative interviews.

**Results:**

Based on combined thematic analysis of 59 transcripts, we identified two major themes: 1) Integration: patients and professionals valued collaborative ways of working because it enhanced co-ordination of mental and physical health care and provided a sense that patients’ health was being more holistically managed. 2) Division: patients and professionals articulated a preference for therapeutic and spatial separation between mental and physical health. Patients especially valued a separate space outside of their LTC clinic to discuss their emotional health problems.

**Conclusion:**

The COINCIDE care model, that sought to integrate depression care within the context of LTC management, achieved service level integration but not therapeutic integration. Patients preferred a protected space to discuss mental health issues, and professionals maintained barriers around physical and mental health expertise. Findings therefore suggest that in the context of mental-physical multimorbidity, collaborative care can facilitate access to depression care in ways that overcome stigma and enhance the confidence of multidisciplinary health teams to work together. However, such care models need to be flexible and patient centred to accommodate the needs of patients for whom their depression may be independent of their LTC.

## Background

Reducing the burden of depressive disorders is recognised as a major public health priority [1]. Depression is common in patients with long term conditions (LTCs), and patients with comorbid depression and LTCs have significantly greater reductions in health status compared with patients with depression alone [2]. Additionally, the coexistence of depression and LTCs is also associated with increased mortality and unscheduled care, with significant cost implications [3]. However, despite the availability of effective therapies for depression in people with LTCs, depression is under recognised and undertreated. In the English NHS, and other advanced health economies, barriers to enhanced care of depression in LTCs can be partly explained by the fact that primary care has traditionally been organised around the delivery of care for single diseases [4]. Furthermore, within the context of primary care, short consultation times and a tendency on the part of patients and practitioners to normalise depression in the presence of LTCs [5] has typically led to prioritisation of physical over mental health problems, thereby limiting opportunities for integrated healthcare. However, the cost and health burden associated with poor management of depression and LTCs has prompted widespread recognition among clinicians [6], policy makers [7], and governments [8] that there are significant gains to be made by developing more integrated ways of working that foster partnership working between mental health and other health professionals [3,9].

In recent years UK health policy for managing LTCs has been informed by US approaches to quality improvement and service redesign: the chronic care model and the ‘risk pyramid’ developed by Kaiser Permanente [10]. These US models are underpinned by a philosophy that appeals to whole system perspectives, in which health care systems are seen as the main barrier to delivering effective treatments for LTCs. Collaborative care, which draws on the chronic care model, is a leading candidate intervention to positively transform the delivery of health care for patients with complex needs, including people with LTCs and depression. Components of collaborative care as described by Gunn and colleagues [11] include:

1. A multi professional approach to patient care (including the use of non-medical case-managers)
2. Structured patient management plans
3. Scheduled follow-ups
4. Enhanced inter-professional communication

When compared with usual care, collaborative care is associated with significant improvement in depression and anxiety outcomes over the short, medium, and long term [12]. However, evidence about the effectiveness of collaborative care is predominantly drawn from trials conducted in the United States, where psychological interventions are only available to patients with health insurance or those who can afford to pay, raising questions about generalisability beyond the US. Furthermore, those US trials which have shown that depression can be improved in people with LTCs using collaborative care not only recruited highly selected populations, but have also relied on elite groups of academic specialists to supervise case managers, thereby limiting the validity of these approaches in more routine settings [13,14]. The CADET trial has since shown the benefits of collaborative care for depression in a UK setting, but again tested a model of care that used academic-specialist supervisors, did not explicitly recruit people with LTCs, and was not embedded in primary care management of LTCs [15].

How best to achieve integrated mental and physical health care in the context of routine primary care is, therefore, still largely unknown. Integrated management of comorbid mental and physical problems may be particularly complicated given the perceived reluctance of patients with LTCs to acknowledge depression [5] and the complexity of patient responses to depression in the context of chronic physical illness [16]. For example, a naturalistic pilot study of collaborative care for patients with LTCs and depression in the UK found that traditional professional divisions perceived between mental and physical health problems remained despite a focus on integration, but patient perceptions were not explored [17].

COINCIDE is a large UK pragmatic trial of collaborative care which tested whether depression could be improved in people with LTCs by integrating low-intensity psychological interventions within the context of routine primary care management of LTCs [18]. This trial involved training low-intensity psychological therapists (Psychological Well-being Practitioners - PWPs) employed by the Improving Access to Psychological Therapies (IAPT) service to deliver psychological therapies to patients with physical-mental comorbidities and to act as case-managers. The treatment plan included two joint meetings between PWPs and practice nurses (PNs). These joint sessions were designed to enhance integration of mental and physical healthcare through improved inter-professional communication and increased opportunities to tailor depression treatments to meet the needs of patients with LTCs. Each patient was offered up to 8 sessions with the PWP over 12 weeks. The sessions involved a biopsychosocial assessment, exploration of the links between their conditions, and active treatment using a goal-oriented psychological intervention to address mental health symptoms. The specific treatment was chosen based on patient preference, and could involve guided self help, behavioural activation, graded exposure, cognitive restructuring and/or lifestyle advice. PWPs received 1 week of training from a multidisciplinary team and received 1 hour of supervision per week from their IAPT senior manager.

This paper reports the results of the nested qualitative study within the COINCIDE trial which aimed to examine:

- How the collaborative care model was implemented by usual care providers in a UK setting.
- How patients and providers understood and experienced the integration of mental and physical health care.

Findings are used to inform the discussion about the development and implementation of the next generation of collaborative care models for managing mental-physical multimorbidity.

## Methods

### Study design

Qualitative methods were chosen to explore perceptions about the model from participants’ own viewpoint. Semi-structured interviews were chosen to enable unrestricted focus on the research question with freedom to explore emerging issues with participants. Interviews were conducted by two research assistants, health service researchers trained in qualitative methodologies.

Ethical approval was granted by National Research Ethics Service Committee North West – Preston (NRES/11/NW/0742)

### Sampling and recruitment

**Figure 1:**
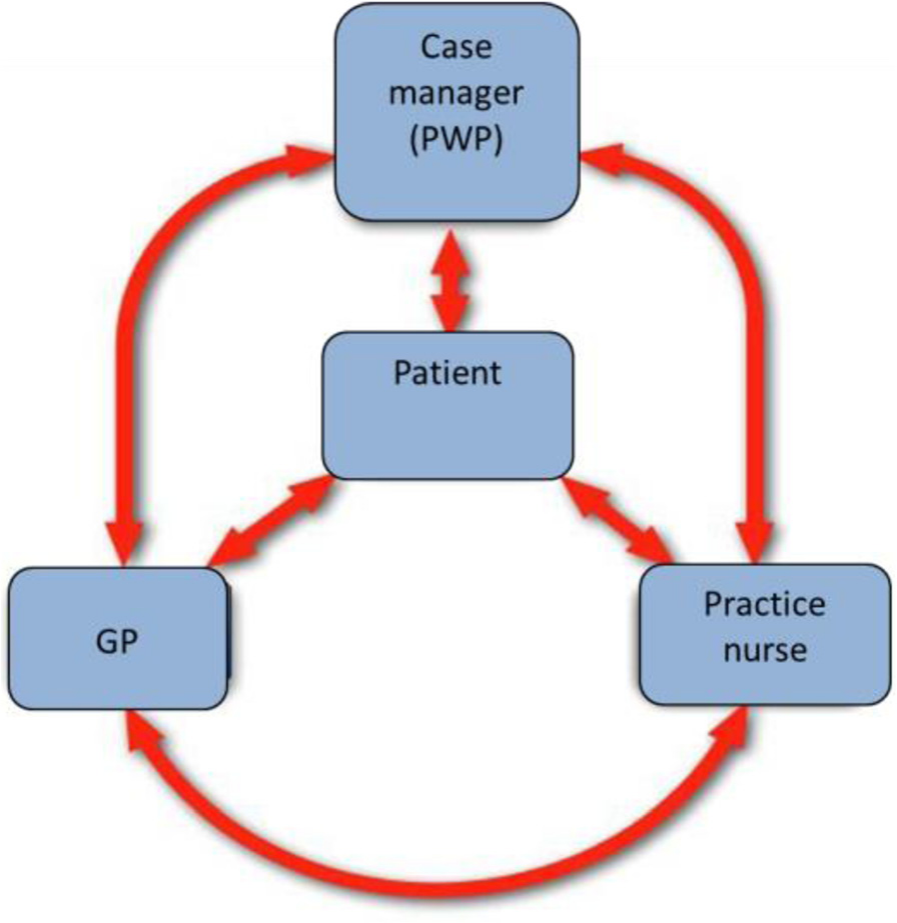
**Case management in the coincide trial.**

The model of collaborative care employed in the trial involved a PWP, PN and GP (see Figure 1). All PWPs, PNs and GPs in the intervention arm of the trial (and so who had been involved in delivering collaborative care) were approached for interview. 11 PWPs, 12 PNs, and 7GPs were interviewed from 15 practices from a total of 17 practices in the intervention arm of the trial across the North West. All PNs were women and Registered General Nurses, with two Nurse Prescribers. The PWP sample consisted of 7 PWPs (4 women, 3 men) and 4 senior PWPs (3 women, 1 man, where senior refers to PWPs in a management/supervisory role) The GP sample comprised 4 men and 3 women.

Patient participant details are presented in Table 1. We sought to identify patients who had completed treatment and also those who dropped out or disengaged in order to capture a spectrum of experiences. 15 patients who completed treatment and 16 patients who disengaged from treatment agreed to be interviewed. ‘Disengaged’ refers to patients not completing all their allotted treatment sessions with the PWP, and so withdrawing from treatment early (rather than by agreement with the PWP). Our intention was to examine whether disengagement was associated with any particular barriers to delivering or receiving the intervention.

**Table 1:** **Patient characteristics**

**ID**	**Gender**	**Completed/disengaged**	**Age**	**Diagnosis of CHD or diabetes**	**Other conditions**
PT01	Male	Disengaged	69	Diabetes	Arthritis, Dry Macular Left eye/Wet Macular right eye, Deafness, Acoustic Neuroma
PT02	Female	Disengaged	85	CHD and Diabetes	
PT03	Female	Disengaged	68	CHD	
PT04	Female	Disengaged	58	CHD	
PT05	Male	Disengaged	59	Diabetes	
PT06	Female	Completed	56	Diabetes	High Blood Pressure
PT07	Male	Disengaged	65	CHD	
PT08	Female	Completed	53	Diabetes	Migraine, High Blood Pressure
PT09	Female	Completed	29	Diabetes	
PT10	Female	Completed	53	CHD and Diabetes	COPD, Cataract, Asthma
PT11	Female	Completed	78	CHD and Diabetes	Facet joint degenerative condition lumbar region, High Blood Pressure
PT12	Male	Completed	60	CHD and Diabetes	Arthritis
PT13	Female	Completed	61	Diabetes	
PT14	Female	Disengaged	73	Diabetes	Heart Murmur
PT15	Male	Completed	43	CHD	Acid reflux, Sinusitis, Asthma
PT16	Male	Disengaged	77	CHD	Asthma
PT17	Male	Disengaged	58	CHD	
PT18	Male	Disengaged	73	Diabetes	Polymyalgia rheumatica, Asthma
PT19	Male	Completed	72	CHD	
PT20	Male	Completed	49	Diabetes	
PT21	Male	Completed	65	CHD and Diabetes	
PT22	Male	Disengaged	64	CHD	COPD, Sleep Apnoea, Arthritis
PT23	Male	Disengaged	56	Diabetes	
PT24	Female	Disengaged	52	Diabetes	Vasculitis, Osteoporosis, Hyper parathyroidism, Kidney failure
PT25	Male	Disengaged	60	Diabetes	Arthritis
PT26	Female	Completed	56	Diabetes	
PT27	Male	Completed	65	CHD	Multiple Myeloma
PT28	Male	Disengaged	58	Diabetes	
PT31	Male	Disengaged	46	CHD	

### Data collection

61 individual interviews were completed between October 2012-October 2013 until concurrent analysis indicated that theoretical saturation had been reached. Interviews were conducted by two researchers trained in qualitative methods. All patients were interviewed after their first follow up (4 months after recruitment) and all interviews were completed before the trial ended. Topic guides were developed based on study aims and informed by a previous qualitative study conducted during the pilot phase of the trial [17], and also developed iteratively as the study progressed. Professional topic guides explored participants' perceptions about what collaborative care is and its value (eg. “What do you understand by ‘collaborative care’?”), and perceptions of priorities and challenges in treating physical-mental comorbidities (eg. “What are the challenges of treating a people with physical-mental comorbidities? How much is mental health care prioritised or not when treating a long term condition?”). They also explored experience of delivering and implementing the model, for example, asking about who was involved, whether the model changed the way they worked or how care was delivered, and whether they felt it had impacted on patients. Patient topic guides began by exploring participants’ condition and previous experience of treatment (eg. “Can you tell me a bit about your health condition?”, “Have you talked about depression with your nurse or GP before?”), their perception of the intervention, such as their expectations for treatment and their relationship with the health professionals, whether the mental health support offered was appropriate for people with physical LTCs, and whether they found the treatment beneficial. Patients who did not complete treatment were specifically asked why they did not continue and barriers to engagement explored.

### Data analysis

Analysis of the data was completed before the results of the COINCIDE trial were known and was therefore completed blind to study outcomes. Transcripts for two participants (PT29 and PT30, both completers) were lost due to recording error and analysis was completed on the remaining 29 patient transcripts and 30 professional transcripts. All transcripts were read by two authors (SK, IA) and a subset read by the other authors. Analysis was guided by the principles and procedures of the constant comparative method [19,20]. There was inductive initial coding of text segments, followed by re-coding and memo writing in order to generate conceptual themes. Consensus meetings between the study team then discussed and agreed on overarching thematic interpretations. We actively sought disconfirming comments but found a strong consensus around the apparent themes. Patient and professional transcripts were analysed independently in the first instance but an integrated analysis was conducted when it became apparent that the professional and patient data sets produced a more comprehensive and illuminating account when considered concurrently rather than as distinct data sets.

## Results

The data can be understood using two over-arching major themes, which were distinct but complementary in illuminating how collaborative care was implemented and perceived by both patients and professionals. The first theme concerns how the model enabled **integration**, encouraging co-ordination of care for patients’ mental and physical conditions. By contrast, the second theme focused on ***division***, with both patients and professionals emphasising a preference and perceived need for treatment spaces that separated out the management of physical and mental health problems.

### Integration

When asked whether and how collaborative care impacted their clinical work, all health professionals (PNs, PWPs, GPs) emphasised how the new care model had enabled better liaison between themselves and supported signposting patients to a wider range of services. Expressions about “*sharing care*” (PN01), providing the care “*side by side*” (PN10), “*well rounded care*” (PN03), and “*working as a team rather than as individuals*” (GP06) were typical of health professionals’ perceptions about the benefits of integrated working. The framework offered by collaborative care was valued by professionals, especially by PWPs, because it increased opportunities for care co-ordination and information sharing with PNs, and also enhanced their confidence to manage mood problems in the context of complex physical symptoms:

PWP10: *Working collaboratively…in terms of your practice it’s very helpful to get that reassurance that what you’re doing in the sessions is the right thing, is useful, and will be helpful for the person*.

Patients were positive about the enhanced communication between the professionals, who typically had worked in isolation from each other:

PT06 (Female, completed): *Basically the nurse is very good at what she needs to be which is checking things but a lot of it is that she's interested in your physical health… there could be that link so I think both professionals have got to have, you know, like an idea of what the other professional is actually doing and so they're not just working in silence they're working together.*

Consistent with this, health professionals reported an increased awareness of patients’ multiple conditions. The PNs reported that the model helped them understand the patient in a more holistic way, and PWPs valued the broader understanding of the patient that they gained from the additional communication with the nurse.

PN01: *I didn’t realise how much of that was to do with their disease, because generally the people I refer are depressed for some other reason, …and I didn’t put it altogether that, perhaps this is just a whole package of things, it’s not just one thing…So, yeah, it’s made me more aware really of how people think and the one problem they’re presenting with may not necessarily be the only one.*

PWP04: O*ne good thing about the joint meeting with the practice nurse, that it just gave us a lot more information and maybe a lot more background to the patient than what we would normally have in therapy.*

The increased access to and availability of mental health care offered was considered much needed by both PNs and patients, who reported that routine management by the nurses was typically restricted to focusing on LTCs with no space to discuss their mental health problems, particularly the ‘everyday’ struggles of living with depression and chronic illness:

PT24 (Female, disengaged): [*The GP and PN] haven’t got the time. No, it has to be people that are trained listeners and also have the time that they can devote to a session like that. I think there are counselling sessions but I think you’ll find it’s full of people with schizophrenia and that sort of depression, rather than us who just plod on, feeling very blue, not achieving as much as we could and not feeling our best, but we’re not going to kill ourselves tomorrow. I think the service as it is now could only cope with crises, rather than helping the everyday person, which is probably a much larger number of people, isn’t it?*

Both PNs and PWPs also suggested that the collaborative care framework facilitated delivery of mental health care in a more acceptable, less stigmatised way. In this sense embedding mental health care in the context of patients’ physical health problems increased the accessibility of the mental health treatment for this patient group, as patients could work with PWPs without necessarily characterising their problems as ‘depression’:

PN10: *If you mention mental health there is still that - yeah, another word for it is it’s still a stigma. People don’t like to address it, admit to it, or whatever. So maybe I think it needs to be addressed side by side as part of the whole care. At present it does, anyway, until people’s attitudes change.*

PWP10: *I had one person who wouldn’t even say the words, anxiety and depression, he didn’t see himself as being anxious or depressed at all, so we didn’t use those words. He still engaged, and still did the work, but we just kind of skirted round those words, yes! And he still really felt the benefits of it, and was really a different person when he finished.*

The integrated working was emphasised to be between PWPs and PNs, with GPs having relatively little involvement, consistent with other studies of collaborative care in the UK [21]. Patients themselves felt GPs would be unable to contribute due to time restrictions and preferred the closer involvement of the PN and the low intensity PWP.

### Division

So far we have shown how a collaborative care model that integrated depression care within primary care of LTCs was valued by PWPs, PNs and GPs because it facilitated greater coordination of mental and physical care and also enhanced their confidence to manage patients with a complex mix of physical and mental health problems. For their part, patients’ perceptions of what integration meant revolved around the sense that the collaborative approach granted them access to mental health care that had hitherto been out of reach, either because physical health problems had taken centre stage in routine primary care consultations or because seeking mental health treatment was stigmatised.

We now show that *service level* integration in the context of collaborative care for depression and LTCs did not necessarily equate to *therapeutic* integration. Indeed both PWPs and PNs maintained explicit role divisions around delivering mental and physical health care, often drawing on a narrative about the limits of their expertise:

PN10: *We see patients in primary care and try to be holistic, [but] we have to realise that we do have limitations in what care we can provide and sharing patient care with other professionals…You have to realise that you have limitations and there comes a point where there are other better qualified people who are better able to care for that patient.*

PWP04: *I think, you know, as I say, my area is obviously mental health, and her area was more physical health … So there was no real, you know, crossover*

This separation between what constituted physical and mental health care was reinforced by patients as well. In part, separation was seen by patients as a natural by-product of health professionals’ expertise, but it was also linked to treatment preferences. While patients recognised the value of seeing PWPs in the same geographic space as their nurse, (as co-location was seen to enhance care coordination and removed the stigma of accessing mental health treatment), they often stated a preference for discussing emotional health problems in a separate therapeutic space away from the nurse:

PT20 (Male, Completed): *its two different things. I wouldn’t go to [PN name] and start crying my eyes out and saying I miss my dad and all that. She controls my medication. That [the mental health aspect] was emotional…Separate. Absolutely separate… I don’t think you’re ever going to get one person doing all that.*

PT12 (Male, Completed): *[The PWP is] more qualified in that sense [talking about emotions]. She’s… the nurse basically looks after your body, not your mind. Each one’s got a job to do.*

Patients’ perceptions about therapeutic integration were also shaped by their experience of the joint consultation meetings between the PNs and the PWPs. These joint meetings may have reflected a sense of joined up working on the part of professionals and led patients to feel confident that their care was being more appropriately and expertly managed, but they did not lead to care that sought to treat both their physical and mental health problems synergistically. In fact for some patients the joint consultation meetings were deemed to be unnecessary and just about “*comparing notes*” (PT20), and several commented that it appeared to be more useful for the professionals than for patients. Patients appreciated that the professionals were now ‘working together,’ but with clear divisions in the work undertaken still emphasised:

PT21 (Male, Completed): *Knowing somebody is depressed is a good idea, but I don’t think it’s [the nurses] job to…because as I say they’re medical people, they treat people come in with their toenails and whatever…. I think it’s a good idea that they should know that you’ve got a bit of depression because when I go in there and she says your blood sugar, I said well, I’ve been a bad boy, I’ve eaten this, that and the other, she shouldn’t start saying, oh, what are you doing that for?!*

GPs and nurses were cast as ‘insiders’ who knew their patients and had responsibility for ‘controlling’ their medical conditions, whereas PWPs were cast as ‘outsiders’ which paradoxically granted patients freedom to talk emotionally about their life circumstances and medical conditions in ways that were not possible with GPs and nurses:

PT13 (Female, Completed): *You do stop and you think to yourself how am I going to cope with it, but you don’t have anyone to say that to. You don’t have a professional to say that to … I mean I’m not saying a GP’s not qualified to do that. I’m sure they are. But at the same time they’re the ones who are controlling your condition…. I think with [PWP name], it is like because he’s an outsider, because he just let me talk.*

In fact, attempts to explicitly integrate physical and mental health treatment were resisted by patients when it encroached on their freedom to talk about other factors, outside of their physical health, that might be linked to their mental health. Consistent with the previous excerpts, patients wanted the mental health treatment to be separate and distinct from their physical health management, and struggled with sessions that focused on dealing with their mental health condition only in the context of their physical illness.

PT03 (Female, Disengaged): *When [the PWP] was asking me questions … he wanted me to say that I felt very depressed over my heart trouble, and that, and I didn’t and I couldn’t say I did, because it would have been dishonest*

PT24 (Female, Disengaged): *I think that was the problem for [the PWP], she kept coming back to just diabetes; now, we’re just talking about the diabetes, how does that affect you? If they could look at the wider issue, yes, it’s brilliant and it’s well needed for most people that are chronically ill.*

These tensions between integration and division were keenly felt by health practitioners who attempted to implement the COINCIDE care model as per the trial protocol i.e., as an integrated psychological intervention for depression for people with LTCs. Both PWPs and PNs reflected on instances where they started with an integrated treatment philosophy but were often led away from a focus on LTCs by patients who valued a less specific discussion about the genesis and maintenance of their mood disorder.

PWP06: *We tried to do a gated, boundaried piece of work… Some of them wanted to receive treatment on their mental health and talk about things that were nothing to do with their health condition*

PWP02: *Sometimes they [patients] will go off on a tangent so to speak and start to talk about other things… but it’s not so relevant in terms of the physiological condition that we had on the trial…it could be something that’s happened years ago and they’ll want to talk about that, but it’s not really relating to diabetes or chronic heart disease.*

PN02*: They thought it was to bring everything else in as well …. a lot of the issues weren’t just related to their long term condition. Their depression was related to things that were going on in their family life which was nothing to do with that actual condition, it was social things… we have to stick to what we were wanting to get out of this study when it was nothing to do with them*

Notably this may have been an artefact of the trial (as the professionals refer to ‘the trial’ and ‘the study’), but it may also relate to the well documented prioritisation of physical health at the expense of mental health:

PN04: *I think if you asked a patient what their agenda was a lot of patients would say, yes, the depression is outweighing everything else, but obviously for the healthcare point of view sometimes you look at results, and you have to put it holistically with the patient, you know, and think, golly, these results are diabolical, we’ve got to get your diabetes on track, and then the depression would take a second seat I think really*

Some patients reported valuing the opportunity to discuss the emotional impact of their health conditions, but the data suggest that preference for this was varied, and patients who wanted to talk about issues beyond their LTC were not always given the desired space to do so. The joint meetings, which attempted to directly integrate the mental and physical health care received were consequently uncomfortable for some patients:

PT01 (Male, Disengaged): *In many ways I was dreading it, because, I just couldn’t see the point in it. I thought it was just an embarrassment. An embarrassment for the nurse and embarrassment for me. We didn’t have anything to say.*

This further supports the interpretation that patients valued access to mental health care outside of their typical LTC management, and with a professional not involved in their physical care. Explicit attempts to integrate mental and physical health care within joint therapy sessions was considered inappropriate or irrelevant, and potentially undermined the patient’s need for their mental health condition to be independently valued and explored.

## Discussion

This study explored professional and patient perceptions of what integrated care meant in the context of collaborative care for people with depression and multimorbidity. The data reveal how the collaborative care framework was valued by professionals for providing greater co-ordination between different specialities and increasing their confidence that all aspects of the patient's condition were appropriately managed. Different attitudes and approaches between mental health specialists and generalists in primary care have been referred to as the ‘covert barriers’ to integration [22] but we found here that expertise and difference were not a source of tension between PNs and PWPs. Indeed, in the context of collaborative care, mental health professionals achieved parity of esteem with their nurse colleagues owing to their recognised expertise in mental health. Equally, patients valued therapeutic boundaries between the delivery of health care for physical and mental health problems, as this maintenance of separate therapeutic spaces liberated discussion of emotional problems that would have ordinarily been relegated to the margins in routine LTC clinics. However, for patients, joint consultations proved to be a more contested and less useful therapeutic space. Patients reported that these joint sessions appeared more useful to professionals and indeed attempts to explicitly integrate mental and physical treatments were largely unsuccessful – they undermined patient experience and satisfaction and possibly reasserted historical tensions in primary care by focusing on physical rather than mental health needs.

### Comparison with other studies

There is evidence that primary care nurses are possibly best placed to act as case managers in collaborative care models, suggesting that integration of mental and physical mental health care can be achieved without the need for bringing mental health providers into primary care. A recent meta-analysis of 14 randomised controlled trials (n = 4440) of nurse-led collaborative care reported moderate effects on depression severity in people with LTCs (standardised mean difference 0.43 95% CI 0.34 to 0.52) when compared with usual care, suggesting that nurses are best placed to manage depression in people with LTCs [23]. However the generalisability and feasibility of such a model is open to serious scrutiny. Of the 14 studies included in the meta-analysis by Ekers et al., 11 were from the United States, and the one UK study included was based in a specialist cancer setting casting doubt about the relevance of findings to primary care settings outside the US. Furthermore one of the trials included in this review only achieved improvements in both physical and mental by implementing separate rather than integrated treat to target protocols for depression and diabetes/CHD [24].

Evidence drawn from an updated service evaluation of telephone delivered nurse case management for depression in one rural region of the English NHS has similarly supported the use of nurses as case managers. This service evaluation showed that among 218 patients drawn from 13 general practices, nurse case management was associated with a mean reduction of 8.9 points on the PHQ-9 five years after the nurses were originally trained; mean change in depression severity was similar in a sub-group analysis of 37 patients with long term conditions [25]. However, feasibility and sustainability of nurse-led collaborative care for depression and LTCs was particularly questionable in this context given that only three of the 13 nurses originally trained in this collaborative care service evaluation had continued to deliver the interventions beyond the initial study period, with time constraints, budget cuts, and the emotionally draining nature of case management cited as reasons why nurses had stopped delivering the interventions. Even nurses who had continued to deliver collaborative care voiced concerns that they felt less skilled to manage mental health problems compared with physical problems [25]. These reflections resonate with other qualitative work with nurses with responsibilities for delivering psychological interventions for patients with LTCs, which has shown that nurses can find such work challenging and ‘qualitatively different’, raising concerns over competence [26]. Our study would support the notion that collaborative care for depression and LTCs is more likely to be successful and implementable when delivered by a coordinated and well supervised team of recognised experts in mental and physical health rather than by nurses alone.

Returning to the original premise made by Wagner et al [27], our study demonstrates that the value of the collaborative care lies mainly in the reorganisation of treatment *delivery* to support people with long term conditions to access and engage with mental health services. Patients in this study were unclear of the value or need for more directly integrated treatment, apparently due to the perceived distinctness of mental and physical health problems. This contrasts to current initiatives focusing on minimally disruptive medicine which emphasise harmonisation of treatment approaches to reduce the burden on patients with multimorbidity [28], possibly through development of ‘synergistic’ treatment models [29]. Delivering care that is consistent with patient preferences is likely to be a key driver of the sustainability of collaborative care in practice [30], and our study demonstrates the importance of exploring preferences in the context of physical and mental co-morbidities which may have novel implications for management of multimorbidity.

Critically, our study suggests that patients with depression and long term conditions require flexible models of care that support access to psychological treatments delivered by recognised experts, rather than overly complex integrated care models that seek to tie treatment of depression to treatment of physical illness. Although some patients did wish to explore the emotional impact of their LTC, consistent with other studies patients wished to explore wider social or psychological issues [31] and valued the mental health sessions precisely because they were ‘outside’ the management of their LTC. They may have particularly appreciated the opportunity to do this given that practice nurses have been shown to lack confidence in dealing with psychosocial aspects of depression in physical health [32], and psychosocial issues may otherwise be neglected in traditional management models.

The data also suggest that closely linking physical and mental health care can inadvertently undermine the mental health treatment as physical management can become privileged; instead patients require a more open and flexible approach rather than presuming a preference for therapeutic integration. The collaborative model tested in COINCIDE that embedded low-intensity psychological therapists employed and supervised by IAPT in primary care provides a template for such a model. Furthermore, this model could be scaled up and spread at pace because its reach is not overly reliant on the input of academic specialists or limited by time constraints imposed on nursing staff, and consequently it may be a more feasible and acceptable model to implement into routine care.

### Limitations

This study systematically explored patient and provider perspectives within the context of a pragmatic trial of collaborative care, making the evaluations more aligned with those found in a natural experiment in routine settings. However, there are several limitations that must be considered. Firstly, although COINCIDE adopted a pragmatic approach to approximate routine care conditions, our evaluation necessarily reflects how the intervention was delivered and experienced within the structure of a controlled trial. Secondly, we only evaluated the impact of the care model over the short term and the long-term implications of embedding IAPT workers in primary care for both patients and providers are unknown. Sustainability of the COINCIDE model in particular may be better understood through longitudinal evaluations that use mixed-methods under more naturalistic conditions. Thirdly, although the older patient sample included here is representative of the population seeking help for mental-physical comorbidities, the preference for division in mental and physical health treatment should not be assumed to apply to patients from other generations. Finally, the findings may not generalise to contexts where collaborative care models will rely on different professionals, in particular cases where the GP/family doctor is more involved in provision of mental health care [33], and in countries where low-intensity psychological workers are not available to be co-located within primary care settings.

## Conclusion

There have been sustained calls for the next generation of collaborative care trials to focus on tests of implementation rather than tests of effectiveness [34,35]. Large reviews can struggle to identify how individual components of complex interventions like collaborative care contribute to its impact [36]. Qualitative studies, particularly those nested within large scale evaluations such as COINCIDE, can both draw out which aspects of the model are perceived as valuable to patients and providers, and also help to identify and characterise barriers and enablers to implementing collaborative care in routine care. Our study demonstrates that for complex patients with physical-mental multimorbidity, collaborative care is best understood as a way of organising services to meet distinct physical and mental health needs, not least the need to increase access to psychological therapy and provide a much-needed space for patients to explore their mental health problems outside of their routine LTC management. Therapeutic integration was not always necessary or valued and patients preferred their mental and physical health to be managed by recognised experts, not just by their primary care nurse. The development of pragmatic and flexible models of collaborative care that meet the needs of patients with physical-mental multimorbidity is therefore needed to meet the policy objectives of “No Health Without Mental Health” [8] in practice.

## Abbreviations

LTCs: Long term conditions
IAPT: Improving access to psychological therapies
PWP: Psychological wellbeing practitioner
PN: Practice nurse
CLAHRC: Collaboration for leadership in applied health research
NIHR: National institute for health research
